# The Structure of the EU Mediasphere

**DOI:** 10.1371/journal.pone.0014243

**Published:** 2010-12-08

**Authors:** Ilias Flaounas, Marco Turchi, Omar Ali, Nick Fyson, Tijl De Bie, Nick Mosdell, Justin Lewis, Nello Cristianini

**Affiliations:** 1 Intelligent Systems Laboratory, University of Bristol, Bristol, United Kingdom; 2 Joint Research Centre (IPSC), European Commission, Ispra, Italy; 3 Bristol Centre for Complexity Sciences, University of Bristol, Bristol, United Kingdom; 4 School of Journalism, Media and Cultural Studies, Cardiff University, Cardiff, United Kingdom; Fondazione Telethon, Italy

## Abstract

**Background:**

A trend towards automation of scientific research has recently resulted in what has been termed “data-driven inquiry” in various disciplines, including physics and biology. The automation of many tasks has been identified as a possible future also for the humanities and the social sciences, particularly in those disciplines concerned with the analysis of text, due to the recent availability of millions of books and news articles in digital format. In the social sciences, the analysis of news media is done largely by hand and in a hypothesis-driven fashion: the scholar needs to formulate a very specific assumption about the patterns that might be in the data, and then set out to verify if they are present or not.

**Methodology/Principal Findings:**

In this study, we report what we think is the first large scale content-analysis of cross-linguistic text in the social sciences, by using various artificial intelligence techniques. We analyse 1.3 M news articles in 22 languages detecting a clear structure in the choice of stories covered by the various outlets. This is significantly affected by objective national, geographic, economic and cultural relations among outlets and countries, e.g., outlets from countries sharing strong economic ties are more likely to cover the same stories. We also show that the deviation from average content is significantly correlated with membership to the eurozone, as well as with the year of accession to the EU.

**Conclusions/Significance:**

While independently making a multitude of small editorial decisions, the leading media of the 27 EU countries, over a period of six months, shaped the contents of the EU mediasphere in a way that reflects its deep geographic, economic and cultural relations. Detecting these subtle signals in a statistically rigorous way would be out of the reach of traditional methods. This analysis demonstrates the power of the available methods for significant automation of media content analysis.

## Introduction

A trend towards automation of scientific research has recently resulted into what has been termed “data-driven inquiry” in various disciplines, including physics and biology [1]–[5]. The automation of many tasks has been identified as a possible future also for the humanities and the social sciences, particularly in those disciplines concerned with the analysis of text, due to the recent availability of millions of books and news articles in digital format [6], [7].

To date, research into media content has been constrained by two key factors. Firstly, the most common form of analysis is labour intensive, relying upon people to physically examine, interpret or code media content [8], [9]. Even those studies that employ meta-analysis struggle to find large sample sizes and consistent coding frames across the sample of different studies examined [10]. This inevitably limits sample sizes, both in terms of the range of media outlets and the time periods covered. This has become particularly problematic as the range of media outlets has increased. While databases exist for generating larger samples such as LexisNexis which provides a commercial database of news content (available at http://www.lexisnexis.com), the analytical tools they provide are limited. Secondly, scholars are obliged to impose their own analytical frameworks on media content: they can only find those trends or patterns that they already know or suspect are there, formulate very specific assumption about these patterns and then set out to verify if they are present or not. In other words, these investigations are inherently hypothesis-driven.

Automating the analysis of news content could have significant applications, due to the central role played by the news media in providing the information that people use to make sense of the world [11]. Public policy makers, businesses, NGOs and scholars from a wide range of disciplines regularly conduct research to investigate media content. The automation of content analysis is highly desirable, but involves high level tasks such as detection of topic, style and possibly translation. Several systems have been developed for continuous media monitoring like the European Media Monitor (EMM) family of media monitoring applications [12] and the ‘Lydia’ system [13] that have been used for detecting events, spatial and temporal distribution of entities in the news etc, ‘Newsblaster’ [14] and ‘NewsInEssence’ [15] for generating multi-document summarization of news, or even commercial systems like those from Google and Yahoo!. We have a different goal than those media monitoring systems, and that is to detect patterns that can provide some insight of the structure of the European mediasphere. It is worth mentioning that datasets commonly used as benchmarks in text mining works, such as Reuters RCV1/RCV2 [16], are not fully relevant to this project since our work is concerned with combining information from multiple sources.

In this study, we report what we think is the first large scale content-analysis of cross-linguistic text in the social sciences, by deploying various artificial intelligence techniques including recent advances in machine translation [17], [18] and text analysis [19]. We examined the content of media outlets based in the European Union – what we might call the EU Mediasphere - in order to see some of the patterns and trends that emerge. We took advantage of the large scale digitisation of large sections of the mediasphere thereby opening up significant new possibilities for the analysis of global media. Our approach not only allows us to examine huge samples across the mediasphere, but allows the data itself to generate patterns and trends. In other words, this study is largely data-driven and illustrative of the scale and scope of the forms of analysis our approach makes possible.

We analyse 1.3 M news articles in 22 languages proving that the choice of stories covered by an outlet is significantly affected by objective national, geographic, economic and cultural relations among outlets and countries: e.g. outlets from countries sharing strong economic ties are more likely to cover the same stories. We also show that the deviation from average content is significantly correlated with membership to the eurozone, as well as with the year of accession to the EU.

## Results

We selected the top-ten news outlets (established by the volume of web traffic) for each of 27 EU countries that have a presence online and offer their content in the Really Simple Syndication (RSS) feed format. This provides an easy means for machines to automatically collect the content of a web-site. For six countries we found less than ten outlets with appropriate online presence resulting in a set of 255 outlets, and from those we managed to successfully parse a total of 215 for the period of study (Supporting Table S1 contains the list of news outlets). The media we monitored are of various types, notably newspapers and broadcast media. Most of these have off-line versions, that is they are the websites of traditional news media. In order to make them comparable, we used news items that appear in the leading news feed of each – roughly corresponding to the first page, or the main page of the news outlet.

In total we gathered 1,370,874 news items from the top stories of the top outlets of each EU country for a period of six months from August 1st 2009 until January 31st 2010. The non-English language news items (1.2 M) were translated automatically to English by a system based on Moses [18]. Although the method does not create human-quality translations, it gives sufficient quality for our purposes, since the bag-of-words representation we use next is not affected by a possibly incorrect ordering or inflection of the translated words.

The English and the translated news-items were then preprocessed by typical text mining methods that include stop word removal, indexing, stemming and transferral to the TF-IDF bag-of-words space [19]. Any untranslated words are also removed to ensure that the similarity of two articles is not affected by the language in which they were originally written. In order to allow comparable patterns of content to emerge from the data, the news items of each day were linked by content, to form clusters corresponding to the same story. Clustering was made using the Best Reciprocal Hit method, borrowed from the field of bioinformatics [20]. The similarity of two articles was measured using the cosine similarity [19]. Every outlet was hence associated with the set of stories it had covered in the given period.

**Figure 1:**
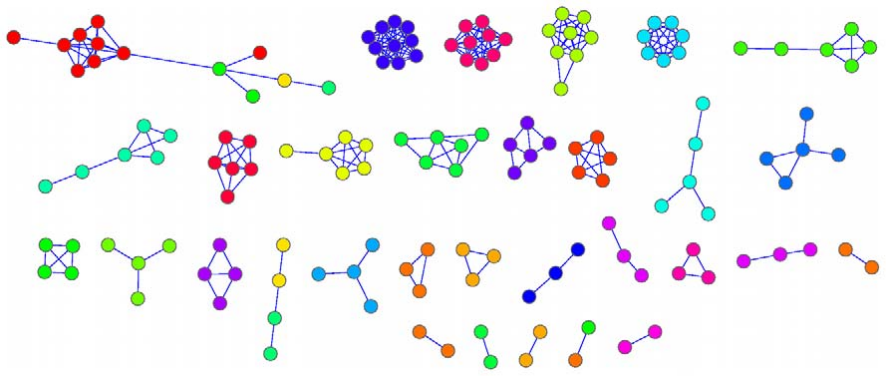
The communities of news outlets in the EU mediasphere. We created the network of the top news outlets per EU country. We connected two outlets if they reported the same stories more than expected by chance as measured by chi-square testing. A high threshold on the chi-square results that maximizes the modularity of the network was used for the current plot. This network is comprised of 147 nodes (outlets) and 263 edges organized in 31 communities. Each outlet is coloured by the country of its origin. Disconnected nodes are omitted. This way the relation of the strongest connected components and countries is revealed.

For each pair of outlets we calculated the chi-square test statistic to measure statistical dependence. A threshold was applied to chi-square values to obtain a network of significantly correlated news outlets. To investigate the underlying structure we applied a high threshold chosen to maximize the modularity [21] of the inferred network. We reached a modularity of 0.93. The resulting network, after removing unconnected nodes, is formed of 31 connected components as displayed in Fig. 1, which were found to correspond roughly to the 27 countries. Given the structure of that network, we calculated the probability that two outlets from the same country end up in the same connected component. This probability is 82.95% and it is significant with p<0.001 as estimated by a randomization test permuting country labels of the outlets (1000 shuffles). The software pipeline starts with the acquisition of 1.3 M articles and ends with the identification of subsets of news outlets that have a strong similarity in the kind of stories they choose to cover. This could seamlessly be applied to much larger datasets.

Given that the network of outlets decomposes into connected components roughly corresponding to EU countries, we have then explored the structure of the network of countries. As for the outlets network, each country is described by the set of stories that appeared in the top-stories feed of the top-outlets of this country. Once more, chi-square scores were used to quantify the statistical independence of the countries in the content of their media. This allowed us to identify similarities and differences between the content of news media in the 27 EU countries, with a resulting pattern across the EU Mediasphere.

We explored three of the many possible factors that may affect the stories that news-media choose to cover and we measured the Spearman correlation of each of these factors to the chi-square scores between countries. The factors we focused on are trade relations between countries, cultural relations and existence of common land borders:

a) For trade relations we used data from United Nations Statistics Division-Commodity Trade Statistics Database, using the total of all trade between the respective countries in 2008. We used the fraction of the total trade of the country in question that is directed towards each other country. We found significant correlation of 31.03% (p<0.001).

b) For cultural relations we used data expressed in the voting patterns of EU countries competing in the Eurovision song contest from 1957 to 2003. We used the fraction of total points awarded by the country in question to each other country over the whole period of time. Countries present in the voting data but not in the current EU countries list were removed prior to normalization. We found correlation of 32.05% (p<0.001).

c) For geographical proximity we used the proportion of length of the common land borders between countries. Correlation was 33.86% (p<0.001).

The predictability of these correlations is important for this study – we would expect patterns of news content to reflect geographic, economic and cultural patterns – since it confirms the ability of our approach to use computer generated means to establish highly plausible patterns in news content.

**Figure 2:**
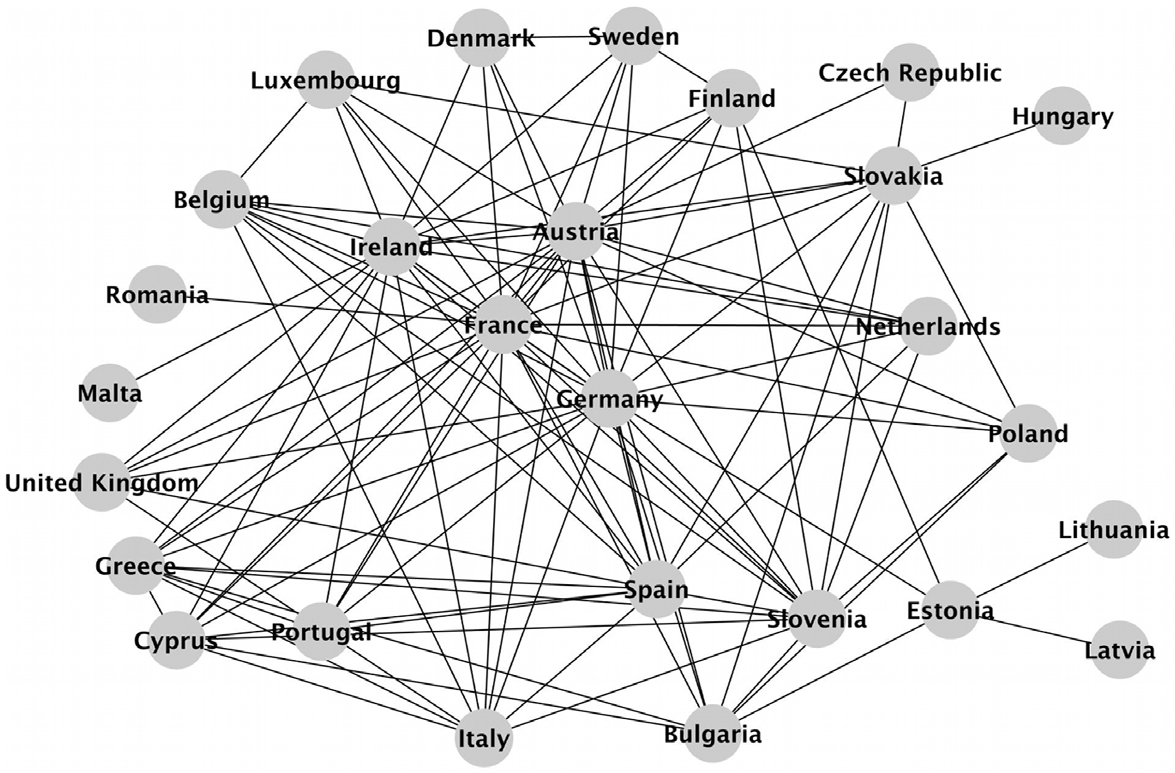
The ‘co-coverage’ network of EU countries. This is the network of the most significant relations among EU countries that cover the same stories in their media. The network has 27 nodes that correspond to the EU countries and 112 links between them. The sparseness was chosen as high as possible with the restriction that all countries must link to at least one other country.

Next, we threshold the chi-square scores between countries to get a network of relations between them. The threshold is chosen as high as possible, while still maintaining a connected network. We call this the ‘co-coverage’ network and we present it in Fig. 2. Several expected connections between countries were found such as Greece-Cyprus, Czech Republic-Slovakia, Latvia-Estonia, United Kingdom-Ireland, Belgium-France etc. Links between countries not explained by borders, trade or cultural relations, could be due to other factors and are potentially the basis of further research from social scientists.

**Figure 3:**
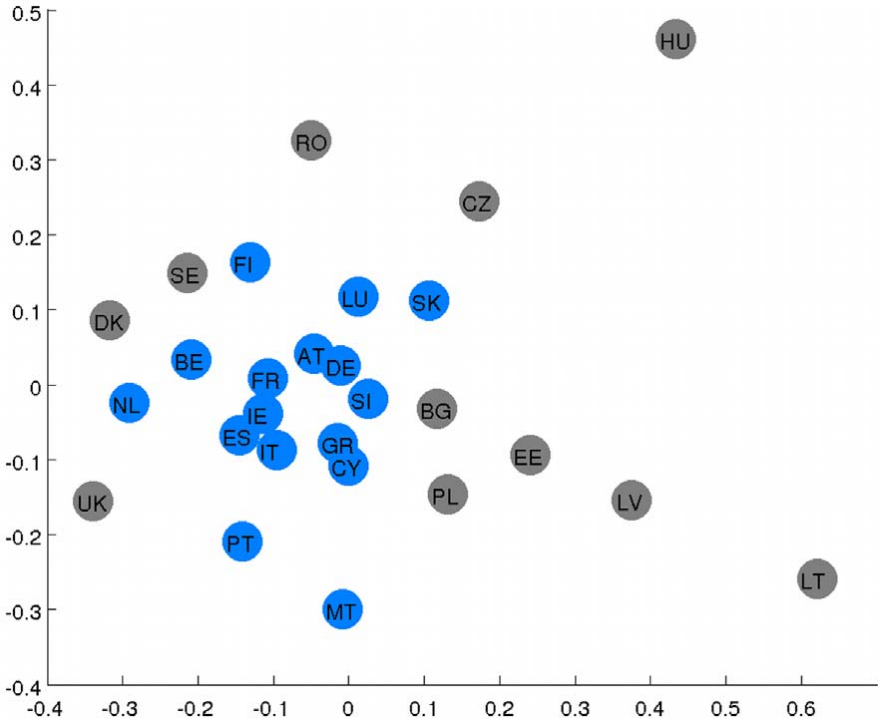
The ‘co-coverage’ map of the EU. This is a 2D representation of the relative positions of EU countries based on their media content. At the centre is the ‘average’ content of the EU media. The Eurozone members are coloured in blue and the rest are coloured in gray. Eurozone countries are closer to each other, and to the average EU behaviour, than non-Eurozone countries.

If we choose not to threshold chi-square scores between countries, we can use non-metric Multidimensional Scaling to treat these scores as similarities and embed them in a 26 dimensional space (N = 27 points projected in N-1 space). In that space, we found the centre of mass of all countries, which represents the average behaviour of news content in the EU, and measured the Euclidean distance of each country from that centre. We found that the ranking of countries based on that distance has a significant (p = 0.0096) correlation of 48.94% to their year of accession in EU (Spearman correlation). As we might expect, on the top of the list are the Eurozone countries presented in Table 1. We can, for example, see that while the UK and Ireland share the same language, news coverage in Ireland appears to be closer to an EU norm than news coverage in the more Eurosceptic UK. By accepting some loss of information we project the countries into a two dimensional space - instead of the 26 dimensional space used previously. In this space, we can reveal their relative positions, where we expect countries that share interest in the same topics to be closer together and those with less common interests in their media content to be further apart. We visualize this in Fig. 3. This illustration presents another insight into the relations between media in different countries of the EU.

**Table 1:** Ranking of countries based on the deviation of their media from average content.

Rank	Country	Euro	A.Year
1	France	Y	1957
2	Austria	Y	1995
3	Germany	Y	1957
4	Greece	Y	1981
5	Ireland	Y	1973
6	Cyprus	Y	2004
7	Slovenia	Y	2004
8	Spain	Y	1986
9	Slovakia	Y	2004
10	Italy	Y	1957
11	Belgium	Y	1957
12	Luxembourg	Y	1957
13	Bulgaria	N	2007
14	Netherlands	Y	1957
15	U. Kingdom	N	1973
16	Finland	Y	1995
17	Sweden	N	1995
18	Poland	N	2004
19	Estonia	N	2004
20	Denmark	N	1973
21	Portugal	Y	1986
22	Malta	Y	2004
23	Czech Rep.	N	2004
24	Romania	N	2007
25	Latvia	N	2004
26	Hungary	N	2004
27	Lithuania	N	2004

## Discussion

We have presented the first large scale comparative analysis of the news content of the leading EU news outlets. We have detected significant patterns and differences in news media content, and have found significant relations between the content of the news media in EU and national, geographic, trade, and cultural relations. This is the first comparative analysis of news content performed on a massive scale, across 27 countries in 22 different languages and over 1.3 M articles, where insights of the structure of the European Mediasphere were obtained, and which was performed largely by automated means. These results could not have been obtained without automated translation and clustering of news, and are just examples of what can currently be achieved in the field of news content analysis by applying automated means of text processing to massive datasets.

Our approach opens up the possibility of analysing the mediasphere on a global scale, using automated means to identify clusters and patterns of content. While this approach inevitably lacks the degree of qualitative subtlety provided by humans, we believe that it is a significant breakthrough in the analysis of media content, allowing for a data-driven approach to social sciences, and the exploitation of the huge amounts of digital data that have become available in recent years.

## Methods

### Selection of Outlets

The selection of the most influential media outlets per country among different types of media outlets is inherently difficult: How can we compare the differences of impact between broadcast and printed media? Since all outlets we study have online presence we used as a measure of their impact the traffic of their websites. This ranking is reported by Alexa.com (Alexa Traffic Rank index) and we collected the rank of each outlet (on May 20, 2009). Similar strategies have been used before in literature, e.g. [22].

### Machine Translation

We applied a Statistical Machine Translation (SMT) approach for translating the non-English articles to English. SMT is based on a noisy channel model [17], where a Markovian Language Model coupled with a phrase-to-phrase translation table are at the heart. In recent years, the noisy channel model has been extended in different directions. The most fruitful has been the phrase based statistical machine translation (PBSMT) introduced by Koehn et al. [23] that is based on the use of phrases rather than words. We use Moses, a complete phrase based translation toolkit for academic purposes. It provides all the state of the art components needed to create a PBSMT system from one language to another. For each language pair, an instance of Moses is trained using Europarl [24] data and JRC-Acquis Multilingual Parallel Corpus [25].

We translated all non-English articles of the following 21 official EU languages into English: Bulgarian, Czech, Danish, Dutch, Estonian, Finnish, French, German, Greek, Hungarian, Italian, Latvian, Lithuanian, Maltese, Polish, Portuguese, Romanian, Slovak, Slovene, Spanish and Swedish.

We make the working assumption that SMT does not alter significantly the geometry of the corpus in the vector-space representation. This is corroborated by results such as [26], a work on cross-language information-retrieval, where bag-of-words representations of translated documents are successfully used.

### Clustering of news articles

News articles were clustered using the Best Reciprocal Hit (BRH) method [20]. An article *i* in outlet *I* is a BRH of article *j* in outlet *J* if a query on outlet *J* with article *i* yields *j* as the top hit article, and the reciprocal query of outlet *I* with article *j* yields *i* as the top hit article. As a similarity measure between two articles we adopted their cosine distance in the bag-of-words space. This results in pairs of articles from different outlets that publish the same stories. Connections of these pairs in larger components is based on the logic that friends of friends are also friends i.e. the pairs of articles *(i, j)* and *(j,k)* form the cluster *(i,j,k)*. The advantages of this clustering method for the current problem are that a) we don't need to specify the number of clusters we want to discover since it is unknown and non-stationary per day and b) we take advantage of the natural separation of the articles into sets of articles published in different news outlets.

### Network Reconstruction

We used an approach based on chi-square test statistic [27] to reconstruct the network of news outlets and observe the underlining structure. The chi-square test measures statistical independence between two variables. In our case the two variables are two outlets and we run the test for each possible pair of outlets. If a test concludes that the two outlets are not independent then we connect them with an edge, otherwise we do not. To measure the chi-square statistic between outlet A and outlet B we count how many stories both outlets published (S_11_), how many stories A published but B didn't publish (S_10_), how many stories B published but A didn't publish (S_01_) and how many stories other outlets published that neither outlet A or B published (S_00_). Then we compute the expected counts that we would have if A and B were independent: E_11_ = (S_11_+S_01_)(S_11_+S_10_)/N where N is the total number of stories, E_10_ = (S_10_+S_11_)(S_10_+S_00_)/N, E_01_ = (S_01_+S_11_)(S_01_+S_00_)/N and E_00_ = (S_00_+S_01_)(S_00_+S_10_)/N. The chi-square statistic of A and B is given by the quantity: 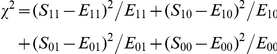



This quantity is associated with the probability that the two outlets being independent. If it is above a threshold we consider A and B dependent and we connect them with an edge. The same approach was used to reconstruct the network of countries.

## Supporting Information

Table S1 The list of media outlets used in research. The table contains the name, the domain name of the online version of the outlet, the country of origin of the outlet, and the RSS feeds that were used.
(0.19 MB DOC)
