# Supplementary material for: The Structure of the EU Mediasphere
Source: PLoS One. 2010 Dec 8;5(12):e14243. doi: 10.1371/journal.pone.0014243 (PMC2999531; doi:10.1371/journal.pone.0014243)
Supplement: Table S1 — The list of media outlets used in research. The table contains the name, the domain name of the online version of the outlet, the country of origin of the outlet, and the RSS feeds that were used. (0.19 MB DOC) [file pone.0014243.s001.doc]

**Supplementary Table**

The list of media outlets used in research. The table contains the name, the domain name of the online version of the outlet, the country of origin of the outlet, and the RSS feeds that were used.

| Name | Domain | Country | RSS |
| --- | --- | --- | --- |
| der Standard | derStandard.at | Austria | http://derstandard.at/?page=rss&ressort=dieStandard |
| diepresse.com | diepresse.com | Austria | http://diepresse.com/rss/ |
| Kleine Zeitung | kleinezeitung.at | Austria | http://www.kleinezeitung.at/klon/rss/news |
| Kronen Zeitung | krone.at | Austria | http://www.krone.at/krone/S25/kmprog/rss.html |
| Kurier | kurier.at | Austria | http://www.kurier.at/newsfeed/nachrichten_nachrichten_rss.xml |
| ORF | orf.at | Austria | http://rss.orf.at/news.xml |
| Salzburger Nachrichten | salzburg.com | Austria | http://www.salzburg.com/nwas/rss.php?channel=7mal24 |
| TT.com | portal.tt.com | Austria | http://portal.tt.com/tt/dt.cms.rss.XMLServer.cls?pub=tt&section=All&CSPCHD=0020000300004kv01fcL000000fJiWirLlKXCrC0wp6dXIEg-- |
| Vorarlberg Online | vol.at | Austria | http://www.vol.at/rss.aspx/page/vol-article-index-rss-page/dc/ |
| WirtschaftsBlatt | wirtschaftsblatt.at | Austria | http://feeds.wirtschaftsblatt.at/c/32352/f/443210/index.rss |
| De Morgen | demorgen.be | Belgium | http://www.demorgen.be/rss.xml |
| De Standaard | standaard.be | Belgium | http://feeds.feedburner.com/dso-front |
| De Tijd | tijd.be | Belgium | http://rss.feedsportal.com/c/32135/f/413342/index.rss |
| GVA | gva.be | Belgium | http://rss.feedsportal.com/c/865/f/413951/index.rss |
| Het Belang van Limburg | hbvl.be | Belgium | http://rss.feedsportal.com/c/865/f/11107/index.rss |
| Het Laatste Nieuws | hln.be | Belgium | http://www.hln.be/rss.xml |
| La Derniere Heure | dhnet.be | Belgium | http://www.dhnet.be/rss/ |
| La Libre Belgique | lalibre.be | Belgium | http://www.lalibre.be/rss/index.xml |
| La Meuse | lameuse.be | Belgium | http://www.lameuse.be/services/fils_rss/actualite/index.xml |
| nieuwsblad.be | nieuwsblad.be | Belgium | http://www.nieuwsblad.be/Rss.aspx?section=Nieuws |
| Actualno.com | actualno.com | Bulgaria | http://xml.webground.bg/actualno/lead/ |
| Bulgaria | bulgaria-weekly.com | Bulgaria | http://bulgaria-weekly.com/index.php?format=feed&type=rss |
| Dnevnik a.m. | dnevnik.bg | Bulgaria | http://dnevnik.bg/rss/ |
| News.bg | ibox.bg | Bulgaria | http://news.ibox.bg/rss_1 |
| Novinite | novinite.com | Bulgaria | http://www.novinite.com/services/news_rdf.php |
| Profit.bg | profit.bg | Bulgaria | http://profit.bg/rss.php?cid=1 |
| Segabg | segabg.com | Bulgaria | http://www.segabg.com/rss/rss20.xml |
| Sofia Echo | sofiaecho.com | Bulgaria | http://feeds.sofiaecho.com/rss2/top-stories |
| Standart | standartnews.com | Bulgaria | http://www.standartnews.com/feeds/rss/ |
| Za Pernik | zapernik.com | Bulgaria | http://feeds.feedburner.com/pernik |
| Ant1 Cyprus | ant1.com.cy | Cyprus | http://www.ant1.com.cy/rss/newsrss.xml |
| Famagusta Gazette | famagusta-gazette.com | Cyprus | http://famagusta-gazette.com/clients/famagusta-gazette/headlines.rss |
| Financial Mirror | financialmirror.com | Cyprus | http://financialmirror.com/get_rss/newsCategory_1/ |
| Phileleftheros | phileleftheros.com | Cyprus | http://www.phileleftheros.com/feeds/cyprus.axd |
| Sigma | sigmalive.com | Cyprus | http://www.sigmalive.com/rss/latest |
| Simerini | sigmalive.com/simerini | Cyprus | http://www.sigmalive.com/rss/pub/9 |
| Typos | typos.com.cy | Cyprus | http://www.typos.com.cy/rss/news.cfm |
| Aktualne | aktualne.centrum.cz | Czech Republic | http://aktualne.centrum.cz/export/rss-hp.phtml |
| Bleskove | bleskove.aktualne.centrum.cz | Czech Republic | http://bleskove.aktualne.centrum.cz/export/rss-bleskove-hp.phtml |
| Czech Happenings | ceskenoviny.cz | Czech Republic | http://www.ceskenoviny.cz/sluzby/rss/index.php |
| Denik | denik.cz | Czech Republic | http://www.denik.cz/rss/z_domova.html |
| Idnes | idnes.cz | Czech Republic | http://servis.idnes.cz/rss.asp |
| Ihned | ihned.cz | Czech Republic | http://ihned.cz/?p=000000_rss |
| Lidovky Noviny | lidovky.cz | Czech Republic | http://www.lidovky.cz/export/rss.asp?c=ln_lidovky |
| Neviditelny Pes | neviditelnypes.lidovky.cz | Czech Republic | http://neviditelnypes.lidovky.cz/export/rss.asp?c=pes_neviditelny |
| Prague Daily Monitor | praguemonitor.com | Czech Republic | http://launch.praguemonitor.com/rss.xml |
| Radio Prague | radio.cz | Czech Republic | http://www.radio.cz/rss/rsscz.xml |
| AOK | aok.dk | Denmark | http://www.aok.dk/forside/feed.xml |
| B.T Online | bt.dk/ | Denmark | http://www.bt.dk/rss/bt/seneste |
| Berlingske Tidende | berlingske.dk | Denmark | http://www.berlingske.dk/section/nyhedsoversigt/&template=rss&mime=xml |
| Borsen | borsen.dk | Denmark | http://borsen.dk/services/newsfeed/rss/ |
| Danish Broadcasting Corporation | dr.dk | Denmark | http://www.dr.dk/OmDR/RSS/20070416101011.htm |
| Ekstra Bladet | ekstrabladet.dk | Denmark | http://ekstrabladet.dk/rss2/?mode=normal&cache300=true |
| fyens.dk | fyens.dk | Denmark | http://www.fyens.dk/rss |
| Jyllands | jp.dk | Denmark | http://jp.dk/rss/topnyheder.jsp |
| politiken.dk | politiken.dk | Denmark | http://politiken.dk/rss/senestenyt.rss |
| Sondagsavisen | sondagsavisen.dk | Denmark | http://www.sondagsavisen.dk/rss.aspx?id=1 |
| Aripaev | ap3.ee | Estonia | http://www.ap3.ee/RSS.aspx |
| Baltic Business News | balticbusinessnews.com | Estonia | http://www.balticbusinessnews.com/RSS.aspx?type=1 |
| Baltic Times | baltictimes.com | Estonia | http://www.baltictimes.com/rss.xml |
| Baltische Rundschau | baltische-rundschau.eu | Estonia | http://www.baltische-rundschau.eu/feed/ |
| Eesti Ekspress | ekspress.ee | Estonia | http://feeds.feedburner.com/EestiEkspressFeed |
| Eesti Paevalehe | epl.ee | Estonia | http://www.epl.ee/?rss |
| LHV | lhv.ee | Estonia | http://www.lhv.ee/images/rss/news.xml |
| Maaleht | maaleht.ee | Estonia | http://www.maaleht.ee/rss |
| Ohtuleht | ohtuleht.ee | Estonia | http://www.ohtuleht.ee/rss.xml |
| Postimees | postimees.ee | Estonia | http://www.postimees.ee/rss/ |
| Aamulehti | aamulehti.fi | Finland | http://www.aamulehti.fi/uutiset/rss.xml |
| Helsingin Sanomat | hs.fi | Finland | http://www.hs.fi/uutiset/rss/ |
| Ilta-Sanomat | iltasanomat.fi | Finland | http://www.iltasanomat.fi/rss/uutiset.xml |
| Iltalehti | iltalehti.fi | Finland | http://www.iltalehti.fi/rss/rss.xml |
| Kauppalehti Online | kauppalehti.fi | Finland | http://rss.kauppalehti.fi/rss/etusivun_uutiset.jsp |
| Nya Aland | nyan.aland.fi | Finland | http://www.nyan.ax/.composer/rss/nyan.rss |
| Sanomalehti Lansi-Suomi | ls24.fi | Finland | http://www.ls24.fi/xml/ls24.xml |
| Satakunnan Kansa | satakunnankansa.fi | Finland | http://www.satakunnankansa.fi/cs/Satellite?c=AMChannelFeed_C&cid=1194596264092&p=1194596263978&pagename=SKA_newssite%2FAMChannelFeed_C%2FLatest10ArticlesRSS20 |
| Taloussanomat | taloussanomat.fi | Finland | http://www.taloussanomat.fi/rss/uutiset |
| Turun Sanomat | turunsanomat.fi | Finland | http://www.turunsanomat.fi/rss/ |
| 20 Minutes | 20minutes.fr | France | http://www.20minutes.fr/rss/flux/une.xml?xts=290428&xtor=RSS-1 |
| Agence France | afp.com | France | http://www.afp.com/english/rss/stories.xml |
| France 24 | france24.com | France | http://www.france24.com/en/monde/rss&language=en |
| France Diplomatie | diplomatie.gouv.fr | France | http://www.diplomatie.gouv.fr/en/backend-fd.php3 |
| La Depeche | ladepeche.fr | France | http://www.ladepeche.fr/rss/39.rss |
| La Provence | laprovence.com | France | http://www.laprovence.com/rss/OM-Actualites-A-la-une.xml |
| La Voix du Nord | lavoixdunord.fr | France | http://www.lavoixdunord.fr/Services/Fils_RSS/France_Monde/index.xml |
| Le Parisien | leparisien.fr | France | http://www.leparisien.com/home/rss/info/rss.xml |
| Liberation | liberation.fr | France | http://www.liberation.fr/rss/laune |
| Radio France Internationale | rfi.fr | France | http://rfi.fr/actufr/pages/001/accueil.xml |
| Derwesten | derwesten.de | Germany | http://www.derwesten.de/nachrichten/nachrichten/politik.rss |
| Deutsche Welle | dw-world.de | Germany | http://rss.dw-world.de/rdf/rss-en-top |
| Focus | focus.de | Germany | http://rss.focus.de/fol/XML/rss_folnews.xml |
| Morgenpost | morgenpost.de | Germany | http://www.morgenpost.de/?service=Rss |
| Spiegel | spiegel.de | Germany | http://www.spiegel.de/schlagzeilen/rss/0,5291,676,00.xml |
| stern.de | stern.de | Germany | http://www.stern.de/standard/rss.php?channel=politik |
| Sueddeutsche | sueddeutsche.de | Germany | http://www.sueddeutsche.de/app/service/rss/ressort/panorama/rss.xml |
| Tagesspiegel | tagesspiegel.de | Germany | http://www.tagesspiegel.de/rss/news.xml |
| Welt | welt.de | Germany | http://www.welt.de/?service=Rss |
| Zeit | zeit.de | Germany | http://newsfeed.zeit.de/wirtschaft/index |
| Eleutheros Typos | e-tipos.com | Greece | http://www.e-tipos.com/rss?category=world |
| Eleutherotypia | enet.gr | Greece | http://www.enet.gr/rss?i=news.el.article |
| ERT | ert.gr | Greece | http://news.ert.gr/xml/rss/rss_2.xml |
| in.gr | in.gr | Greece | http://www.in.gr/rss/feed.asp?FeedSelect=FirstPage |
| Kathimerini | kathimerini.gr | Greece | http://ws.kathimerini.gr/xml_files/worldnews.xml |
| MEGA TV | megatv.com | Greece | http://www.megatv.com/gegonota/rss.asp?catid=2 |
| Naftemporiki | naftemporiki.gr | Greece | http://www.naftemporiki.gr/news/static/rss/news.xml |
| Pathfinder | pathfinder.gr | Greece | http://news.pathfinder.gr/rss/frontpage.xml |
| SKAI | skai.gr | Greece | http://www.skai.gr/rss/rssfeed.php |
| Ta Nea | tanea.gr | Greece | http://www.tanea.gr/default.asp?pid=67&la=1 |
| 168 Ora | 168ora.hu | Hungary | http://www.168ora.hu/static/rss/cikkek_itthon.xml |
| Blikk | blikk.hu | Hungary | http://blikk.hu/static/rss_hirek.xml |
| Budapester Zeitung | budapester.hu | Hungary | http://www.budapester.hu/index.php?option=com_rd_rss&id=4&feed=RSS2.0 |
| Delmagyar | delmagyar.hu | Hungary | http://www.delmagyar.hu/_rss.php?fid=1 |
| Kisalfold | kisalfold.hu | Hungary | http://www.kisalfold.hu/_rss.php?fid=27 |
| Magyar Nemzet | mno.hu | Hungary | http://mno.hu/portal/rss |
| Napi | napi.hu | Hungary | http://www.napi.hu/rssfriss.asp |
| Nepszabadsag | nol.hu | Hungary | http://nol.hu/rss/ |
| Nepszava | nepszava.hu | Hungary | http://www.nepszava.hu/rss/onl_belfold.asp |
| VG | vg.hu | Hungary | http://vg.hu/rss/vg.xml |
| BreakingNews | breakingnews.ie | Ireland | http://feeds.breakingnews.ie/bntopstories |
| Galway Advertiser | advertiser.ie | Ireland | http://feedproxy.google.com/GalwayAdvertiser |
| Irish Examiner | irishexaminer.com | Ireland | http://www.irishexaminer.com/rss/irishexaminer_top_rss.aspx |
| Irish Times | ireland.com | Ireland | http://www.ireland.com/feeds/rss/newspaper/index.rss |
| RTE | rte.ie | Ireland | http://www.rte.ie/rss/news.xml |
| Socialist Worker | socialistworker.org | Ireland | http://socialistworker.org/recent/feed |
| Sunday Business Post | sbpost.ie | Ireland | http://feeds.feedburner.com/tpnews |
| Sunday Tribune | tribune.ie | Ireland | http://www.tribune.ie/feeds/latest/ |
| The Irish Times | irishtimes.com | Ireland | http://www.irishtimes.com/feeds/rss/breaking/index.rss |
| The Limerick Leader | limerickleader.ie | Ireland | http://www.limerickleader.ie/getFeed.aspx?Format=rss&sectionid=3419 |
| ANSA | ansa.it | Italy | http://www.ansa.it/site/notizie/awnplus/topnews/synd/ansait_site_topnews_synd_Today_Idx.xml |
| City | city.corriere.it/ | Italy | http://city.corriere.it/rss/homepage.xml |
| Corriere della Sera | corriere.it | Italy | http://www.corriere.it/rss/homepage.xml |
| Il Sole 24 ORE | ilsole24ore.com | Italy | http://www.ilsole24ore.com/rss/primapagina.xml |
| Kataweb | kataweb.it | Italy | http://news.kataweb.it/feed/hp |
| La Repubblica | repubblica.it | Italy | http://www.repubblica.it/rss/homepage/rss2.0.xml |
| La Stampa | lastampa.it | Italy | http://www.lastampa.it/redazione/rss_home.xml |
| Rai International | rai.it | Italy | http://www.rai.it/dl/portale/html/PublishingBlock-15c2c340-e282-473d-b944-661e818d667b-rss.xml |
| TGcom | tgcom.mediaset.it | Italy | http://www.tgcom.mediaset.it/rss/homepage.xml |
| Virgilio | virgilio.it | Italy | http://notizie.alice.it/generated/rss/rss_topnews.xml |
| Diena | diena.lv | Latvia | http://www.diena.lv/rss/viss.xml |
| Kapitals | kapitals.lv | Latvia | http://www.kapitals.lv/rss/ |
| Latvians Online | latviansonline.com | Latvia | http://latviansonline.com/index.php/rss |
| Latvijas Vestnesis | vestnesis.lv | Latvia | http://feeds.feedburner.com/wwwlvlv |
| LETA | leta.lv | Latvia | http://leta.lv/rss/rss.php |
| Novo News | novonews.lv | Latvia | http://feeds2.feedburner.com/novonews/gTor |
| NRA | nra.lv | Latvia | http://www.nra.lv/rss/index.xml |
| Zemgales Zinas | zz.lv | Latvia | http://www.zz.lv/index.rss |
| Alfa | alfa.lt | Lithuania | http://www.alfa.lt/rss.do?allNews=true |
| Kauno Diena | diena.lt | Lithuania | http://kauno.diena.lt/naujienos/rss |
| klaipeda.diena.lt | klaipeda.diena.lt | Lithuania | http://klaipeda.diena.lt/naujienos/rss |
| Lithuania In Your Pocket | inyourpocket.com | Lithuania | http://www.inyourpocket.com/data/rss/rss_homepage.xml |
| Lithuanian Radio and Television | lrt.lt | Lithuania | http://www.lrt.lt/rss/all.php |
| Litovskij Kurier | kurier.lt | Lithuania | http://www.kurier.lt/rss.xml |
| Lrytas | lrytas.lt | Lithuania | http://www.lrytas.lt/rss/ |
| skrastas.lt | skrastas.lt | Lithuania | http://www.skrastas.lt/?page=rss |
| Vakaru Ekspresas | ve.lt | Lithuania | http://www.ve.lt/rss.php |
| vz.lt | vz.lt | Lithuania | http://vz.lt/RSS.aspx |
| Correio | correio.editpress.lu | Luxembourg | http://correio.editpress.lu/feed/index.1.rss |
| L essentiel | lessentiel.lu | Luxembourg | http://www.lessentiel.lu/rss/front.tmpl |
| Le Jeudi | le-jeudi.editpress.lu | Luxembourg | http://le-jeudi.editpress.lu/feed/index.1.rss |
| Le Quotidien | lequotidien.editpress.lu | Luxembourg | http://lequotidien.editpress.lu/feed/index.1.rss |
| Luxemburg News | topix.com | Luxembourg | http://www.topix.com/rss/city/luxemburg-wi |
| Luxpost | luxpost.editpress.lu | Luxembourg | http://luxpost.editpress.lu/feed/index.1.rss |
| Wort Online | wort.lu | Luxembourg | http://www.wort.lu/wort/web/letzebuerg/luxemburg.xml |
| Int Sabiha Malta Taghna | minnhalsafi.blogspot.com | Malta | http://minnhalsafi.blogspot.com/atom.xml |
| Blogs of the European Commissioners (Malta) | ec.europa.eu/malta-mt | Malta | http://blogs.ec.europa.eu/malta-mt/feed/ |
| Gozo News | gozonews.com | Malta | http://feed.gozonews.com/GozoNews |
| Kullhadd | kullhadd.com | Malta | http://www.kullhadd.com/kullhadd/feed/rss.html |
| Maltamedia | maltamediaonline.com | Malta | http://feeds.feedburner.com/MaltaMediaNews |
| Times of Malta | timesofmalta.com | Malta | http://www.timesofmalta.com/rss |
| Algemeen Dagblad | ad.nl | Netherlands | http://www.ad.nl/?service=rss |
| Dagblad van het Noorden | dvhn.nl | Netherlands | http://www.dvhn.nl/nieuws/index.jsp?service=rss |
| De Gooi-en Eemlander | gooieneemlander.nl | Netherlands | http://www.gooieneemlander.nl/nieuws/index.jsp?service=rss |
| De Telegraaf | telegraaf.nl | Netherlands | http://www.telegraaf.nl/rss/index.xml |
| Haarlems Dagblad | haarlemsdagblad.nl | Netherlands | http://www.haarlemsdagblad.nl/nieuws/index.jsp?service=rss |
| Het Noordhollands Dagblad | noordhollandsdagblad.nl | Netherlands | http://www.noordhollandsdagblad.nl/nieuws/index.jsp?service=rss |
| Leeuwarder Courant | leeuwardercourant.nl | Netherlands | http://www.leeuwardercourant.nl/nieuws/index.jsp?service=rss |
| Leidsch Dagblad | leidschdagblad.nl | Netherlands | http://www.leidschdagblad.nl/nieuws/index.jsp?service=rss |
| NRC Handelsblad | nrc.nl | Netherlands | http://www.nrc.nl/?service=Rss |
| Volkskrant | volkskrant.nl | Netherlands | http://volkskrant.nl/rss/laatstenieuws.rss |
| Dziennik Polska Europa Swiat | dziennik.pl | Poland | http://www.dziennik.pl/?service=Rss |
| Gazeta Olsztynska | gazetaolsztynska.wm.pl | Poland | http://gazetaolsztynska.wm.pl/s/gazeta_olsztynska/rss_gazeta.rss |
| Gazeta Prawna | gazetaprawna.pl | Poland | http://rss.gazetaprawna.pl/GazetaPrawna |
| Gazeta Wyborcza | wyborcza.pl | Poland | http://rss.gazeta.pl/pub/rss/gazetawyborcza.xml |
| Nowa Trybuna Opolska | nto.pl | Poland | http://www.nto.pl/apps/pbcs.dll/section?Category=RSS&mime=xml |
| Puls Biznesu | pb.pl | Poland | http://www.pb.pl/RSS.aspx?topic=aa0a9087-1ce6-4fa2-b2b5-efdf0d68b14c |
| Rzeczpospolita Online | rp.pl | Poland | http://www.rp.pl/rss/2.html |
| Super Express | se.pl | Poland | http://www.se.pl/rss/news/ |
| Thenews.pl | polskieradio.pl | Poland | http://www.polskieradio.pl/iar/wiadomosci/rss.aspx |
| Tygodnik Powszechny | onet.pl | Poland | http://tygodnik.onet.pl/rss.html |
| Acoriano Oriental | acorianooriental.pt | Portugal | http://www.acorianooriental.pt/noticias/rss/ |
| Correio da Manha | correiomanha.pt | Portugal | http://www.correiomanha.pt/rss/default.aspx?channelID=00000009-0000-0000-0000-000000000009 |
| Destak | destak.pt | Portugal | http://feeds.destak.pt/DestakRSS |
| Diario de Coimbra | diariocoimbra.pt | Portugal | http://www.diariocoimbra.pt/index2.php?option=com_rss&feed=RSS2.0&no_html=1 |
| Diario Economico | economico.sapo.pt | Portugal | http://economico.sapo.pt/rss/ultimas |
| Expresso | aeiou.expresso.pt | Portugal | http://aeiou.expresso.pt/gen.pl?p=rss |
| Jornal de Negocios | jornaldenegocios.pt | Portugal | http://www.jornaldenegocios.pt/funcionalidades/rss/generarRSS.php |
| Ojogo | ojogo.pt | Portugal | http://www.ojogo.pt/rss/Noticias.rss |
| Sapo | sapo.pt | Portugal | http://services.sapo.pt/RSS/Feed/noticias/sapo_noticias/diario_de_noticias |
| Sol | sapo.pt | Portugal | http://sol.sapo.pt/rss/ |
| Adevarul | adevarul.ro | Romania | http://www.adevarul.ro/toate-articolele.rss |
| Cotidianul | cotidianul.ro | Romania | http://www.cotidianul.ro/rss.xml |
| Evenimentul Zilei | evz.ro | Romania | http://www.evz.ro/xml/rss |
| Gardianul | gardianul.ro | Romania | http://www.gardianul.ro/stiri_rss.php |
| jurnalul.ro | jurnalul.ro | Romania | http://www.jurnalul.ro/rss |
| Libertatea | libertatea.ro | Romania | http://www.libertatea.ro/rss/index.xml |
| Mediafax | mediafax.ro | Romania | http://www.mediafax.ro/top-news.xml |
| Romania Libera | romanialibera.ro | Romania | http://www.romanialibera.ro/rss/rss.xml |
| Ziarul Financiar | zf.ro | Romania | http://www.zf.ro/rss/zf-english/ |
| Ziua | ziua.ro | Romania | http://feedproxy.google.com/ziua/APBN |
| CAS | cas.sk | Slovakia | http://www.cas.sk/rss/224 |
| Dolezite | Dolezite.sk | Slovakia | http://www.dolezite.sk/rss.php/ |
| eTrend | etrend.sk | Slovakia | http://onas.etrend.sk/trend-holding/rss-sluzba-etrendu/32685.html |
| HN Online | hnonline.sk | Slovakia | http://rss.hnonline.sk/ |
| Noveslovo | noveslovo.sk | Slovakia | http://www.noveslovo.sk/rss.asp |
| Pravda | pravda.sk | Slovakia | http://servis.pravda.sk/rss.asp?o=sk_sph |
| SK Today | sktoday.com | Slovakia | http://www.sktoday.com/rss.xml |
| The Slovak Spectator | spectator.sk | Slovakia | http://www.spectator.sk/articles/rss |
| Ujszo | ujszo.com | Slovakia | http://ujszo.com/rss.xml |
| Delo | delo.si | Slovenia | http://www.delo.si/rss/ |
| Dnevnik | dnevnik.si | Slovenia | http://www.dnevnik.si/rss/ |
| Ministry of Foreign Affairs (Slovenia) | mzz.gov.si | Slovenia | http://www.mzz.gov.si/index.php?id=3256&L=2&type=100 |
| Mladina | mladina.si | Slovenia | http://www.mladina.si/feed/mladina-rss-dnevne-novice.xml |
| Primorske | primorske.si | Slovenia | http://www.primorske.si/rss/ |
| Sinfo | uvi.gov.si | Slovenia | http://www.ukom.gov.si/index.php?id=56&type=100 |
| Slovenska Tiskovna Agencija | sta.si | Slovenia | http://www.sta.si/rss.php |
| Vecer | vecer.com | Slovenia | http://www.vecer.com/rss/ |
| 20 Minutos | 20minutos.es | Spain | http://www.20minutos.es/rss/ |
| Cadena Ser | cadenaser.com | Spain | http://www.cadenaser.com/rss.html |
| El Correo Espanol | elcorreodigital.com | Spain | http://www.elcorreodigital.com/vizcaya/rss/feeds/ultima.xml |
| El Mundo | elmundo.es | Spain | http://rss.elmundo.es/rss/descarga.htm?data2=4 |
| Elpais | elpais.com | Spain | http://www.elpais.com/rss.html |
| Europa Press | europapress.es | Spain | http://www.europapress.es/rss/rss.aspx?ch=94 |
| La Vanguardia | lavanguardia.es | Spain | http://feeds.feedburner.com/lavanguardia/home |
| La Voz De Galicia | lavozdegalicia.es | Spain | http://www.lavozdegalicia.es/portada/index.xml |
| Libertad Digital | libertaddigital.com | Spain | http://rss.libertaddigital.com/libertaddigital/portada |
| Periodista Digital | periodistadigital.com | Spain | http://feedproxy.google.com/PeriodistadigitalcomelPeridicoDeLosPeriodistas |
| Aftonbladet | aftonbladet.se | Sweden | http://feeds.aftonbladet.se/?service=rss |
| Dagens Industri | di.se | Sweden | http://ditrader.di.se/News/rss.aspx |
| Dagens Nyheter | dn.se | Sweden | http://www.dn.se/m/rss/toppnyheter |
| Expressen | expressen.se | Sweden | http://www.expressen.se/1.573280?standAlone=true |
| Goteborgs-Posten | gp.se | Sweden | http://www.gp.se/rss/index.jsp?d=100 |
| Helsingborgs Dagblad | hd.se | Sweden | http://hd.se/?view=rss |
| Metro | metro.se | Sweden | http://www.metro.se/se/rss.xml?c=section-news |
| Svenska Dagbladet | svd.se | Sweden | http://www.svd.se/?service=rss |
| Sydsvenska Dagbladet | sydsvenskan.se | Sweden | http://sydsvenskan.se/?context=xml |
| The Local | thelocal.se | Sweden | http://www.thelocal.se/RSS/theLocal.xml |
| BBC | bbc.co.uk | United Kingdom | http://newsrss.bbc.co.uk/rss/newsonline_world_edition/front_page/rss.xml |
| Channel 4 | channel4.com | United Kingdom | http://www.channel4.com/apps26/syndication/news/itnnews/rss.xml |
| Daily Mail | dailymail.co.uk | United Kingdom | http://feeds.feedburner.com/dailymail/home?in_page_id=1766 |
| Daily Telegraph | telegraph.co.uk | United Kingdom | http://www.telegraph.co.uk/newsfeed/rss/news.xml |
| Guardian | guardian.co.uk | United Kingdom | http://www.guardian.co.uk/rss |
| Independent | independent.co.uk | United Kingdom | http://news.independent.co.uk/index.jsp?service=rss |
| Reuters | reuters.com | United Kingdom | http://feeds.reuters.com/reuters/topNews |
| Sky News | sky.com | United Kingdom | http://news.sky.com/skynews/rss/article/0,,30000-1-100,00.xml |
| The Sun | thesun.co.uk | United Kingdom | http://www.thesun.co.uk/sol/homepage/feeds/rss/article312900.ece |
| The Times | timesonline.co.uk | United Kingdom | http://www.timesonline.co.uk/tol/feeds/rss/topstories.xml |
